# Abnormal Global Brain Functional Connectivity in Primary Insomnia Patients: A Resting-State Functional MRI Study

**DOI:** 10.3389/fneur.2018.00856

**Published:** 2018-11-02

**Authors:** Chao-Qun Yan, Xu Wang, Jian-Wei Huo, Ping Zhou, Jin-Ling Li, Zhong-Yan Wang, Jie Zhang, Qing-Nan Fu, Xue-Rui Wang, Cun-Zhi Liu, Qing-Quan Liu

**Affiliations:** ^1^Department of Acupuncture and Moxibustion, Dongfang Hospital, Beijing University of Chinese Medicine, Beijing, China; ^2^Department of Psychosomatic Medicine, Beijing Hospital of Traditional Chinese Medicine Affiliated to Capital Medical University, Beijing, China; ^3^School of Life Sciences, Beijing University of Chinese Medicine, Beijing, China; ^4^Department of Radiology, Beijing Hospital of Traditional Chinese Medicine Affiliated to Capital Medical University, Beijing, China

**Keywords:** primary insomnia, sleep disorder, degree centrality, functional connectivity, resting-state fMRI

## Abstract

**Background:** Resting-state functional magnetic resonance imaging (fMRI) studies have uncovered the disruptions of functional brain networks in primary insomnia (PI) patients. However, the etiology and pathogenesis underlying this disorder remains ambiguous, and the insomnia related symptoms are influenced by a complex network organization in the brain. The purpose of this study was to explore the abnormal intrinsic functional hubs in PI patients using a voxel-wise degree centrality (DC) analysis and seed-based functional connectivity (FC) approach.

**Methods:** A total of 26 PI patients and 28 healthy controls were enrolled, and they underwent resting-state fMRI. Degree centrality was measured across the whole brain, and group differences in DC were compared. The peak points, which significantly altered DC between the two groups, were defined as the seed regions and were further used to calculate FC of the whole brain. Later, correlation analyses were performed between the changes in brain function and clinical features.

**Results:** Primary insomnia patients showed DC values lower than healthy controls in the left inferior frontal gyrus (IFG) and middle temporal gyrus (MTG) and showed a higher DC value in the right precuneus. The seed-based analyses demonstrated decreased FC between the left MTG and the left posterior cingulate cortex (PCC), and decreased FC was observed between the right precuneus and the right lateral occipital cortex. Reduced DC in the left IFG and decreased FC in the left PCC were positively correlated with the Pittsburgh sleep quality index and the insomnia severity index.

**Conclusions:** This study revealed that PI patients exhibited abnormal intrinsic functional hubs in the left IFG, MTG, and the right precuneus, as well as abnormal seed-based FC in these hubs. These results contribute to better understanding of how brain function influences the symptoms of PI.

## Introduction

The central feature of primary insomnia (PI) is dissatisfaction with sleep quantity or quality, which is associated with difficulty falling asleep, maintaining sleep, or early morning awakening (1). Insomnia is a very common health problem that affects 30 to 35% of adults on an episodic basis and 10 to 12% on a chronic basis (AAoS Medicine^1^)(2). One third or more of the population suffers from a sleep disturbance or excessive daytime sleepiness on a daily basis (3). Insomnia is associated with an increased risk of Alzheimer's disease (4), Parkinson's disease (5), hypertension (6), cardiovascular diseases (7), depression (8), obesity (9), type 2 diabetes (10), and mortality (11). Not surprisingly, insomnia has significant economic and societal impacts. It causes a reduction of $63.2 billion in the total American workforce from the estimated annualized population level due to poor work performance and absenteeism (12). It is crucial to understand the pathologic changes in insomnia by exploring the central nervous system. Findings can offer novel interventional treatments for these patients. However, the etiology and pathogenesis underlying this disorder remain uncertain.

Brain imaging technology has proven to be informative for investigating the central mechanism of PI. Structural neuroimaging studies have revealed brain tissue injury associated with PI by using voxel-based morphometry (VBM) and diffusion tensor imaging analyses (13, 14). Specifically, abnormal regional gray matter volume or white matter integrity have been shown in multiple brain regions, including the anterior cingulate cortex (15), hippocampal (16), medial frontal, and middle temporal gyri (13), thalamus, internal capsule, anterior corona radiate, and corpus callosum (14). The structural changes are usually accompanied by an impairment of brain function. Some studies have reported the abnormal spontaneous functional activity in PI patients by using low frequency fluctuations (17), regional homogeneity (18), and the seed-based functional connectivity (FC) approach (19). Although abnormal structural and functional properties in many brain regions have been found in PI patients, these observations failed to provide information about integrated global brain function alterations. However, the human brain is complex and well organized with coordination of different brain regions as a functional network (20, 21). It is necessary to investigate the brain connectivity within the whole-brain network.

Recently, modern developments in graph theory have delivered important insights into functional brain networks (22). Graph theoretical analysis is a large-scale method and has become an increasingly useful tool to explore the systematic alteration of whole-brain functional organization and connection (23). A graph theoretical analysis based on an automated anatomical labeling atlas study found that chronic insomnia patients showed altered topological characteristics of functional brain networks, expressed as altered nodal in the default mode network, dorsal attention network, and sensory-motor network regions (24). Using graph theoretical analysis based on defined nodes and edges of the networks, previously, it was found that healthy subjects with insomnia symptoms demonstrated reduced regional degree and efficiency in the left inferior frontal gyrus (IFG) compared with healthy subjects without insomnia symptoms (25). Nevertheless, the altered topological properties of the global functional brain network at the voxel level in PI patients are unclear.

Voxel-wise degree centrality (DC) is a graph theory-based and data-driven approach, which can evaluate the importance of each voxel in the brain and every voxel represents its connectivity strength (26). The index of DC is a better connectivity metric than other measurements, because it can assign a higher value to a voxel when this voxel has stronger connections with other voxels in the brain network. Degree centrality emphasizes the impact and significance of a network at voxel level, and it reflects the properties of the functional brain network “hub” in network information communication (27, 28). In a functional brain network, hub regions play pivotal roles in the coordination of information flow (29) and are consistent and stable in healthy human brains but are highly vulnerable to pathological processes (30, 31). Degree centrality measures based on resting-state functional magnetic resonance imaging (fMRI) have been used to observe the alterations of functional networks in diverse diseases, and these measures exhibit relatively high test–retest reliability (32). Focussing on the voxel-wise DC may provide a novel insight into the pathogenesis of PI.

In the present study, DC between PI patients and healthy controls was compared to identify significant alterations in intrinsic functional hubs. Subsequently, we performed further seed-based FC analyses, using the seed regions with significant alterations in the DC analysis, for better detecting the detailed information regarding the connectivity in these hubs. Next, we evaluated the relationships between the clinical features and the DC or FC values. The current study will contribute to the further understanding of the mechanism of PI and provide a bridge for future studies.

## Materials and methods

### Participants

Participants were enrolled from September 2014 through September 2016 at the Beijing Hospital of Traditional Chinese Medicine Affiliated to Capital Medical University. The cohort included 30 patients with PI and 30 healthy controls without insomnia symptoms. Notably, PI patients were recruited in outpatient clinics from the Department of Psychosomatic Medicine, while healthy controls were recruited primarily through advertisements in the community.

The inclusion criteria for PI patients were as follows: (i) age from 25 to 60 years, (ii) right-hand dominance, (iii) meeting the DSM-IV^2^ inclusion criteria for PI, (iv) reporting difficulty in falling asleep, maintaining sleep, or early awakening at the same time for at least 1 month, (v) absence of psychoactive medication use for at least 2 weeks before and during the study. Patients with PI were excluded if they had any of the following: (i) other sleep disorders (e.g., hypersomnia or parasomnia), (ii) insomnia associated with specific reasons such as drugs, alcohol, or physical and mental illness, (iii) history of heart disease, stroke, nephritis, or psychiatric diseases, and (iv) abnormalities in brain structure such as tumors or subdural hematomas.

The healthy controls were age-, sex-, hand dominance-, and education-level-matched to PI patients and were included if they met the following criteria: (i) good sleep quality and a Pittsburgh sleep quality index score <3, (ii) regular sleep habits, (iii) absence of significant heart disease, lung disease, neurological or major psychiatric disorders, (iv) normal conventional brain magnetic resonance imaging (MRI).

Demographics, including age, sex and years of education, of PI patients and healthy controls were collected. Self-rating anxiety scale (SAS), the self-rating depression scale (SDS), Pittsburgh sleep quality index (PSQI), and insomnia severity index (ISI) were also measured. All participants underwent an MRI scan. Four PI patients and two healthy controls were excluded before data analysis because of incomplete MRI data or excessive head motion.

### Standard protocol approvals, registrations, and patient consents

All the study procedures were approved by the Research Ethical Committee of Beijing Hospital of Traditional Chinese Medicine Affiliated to Capital Medical University (reference: 2014BL-003-01). Written informed consent was obtained from participants. All experiments were performed in accordance with relevant guidelines and regulations.

### MRI acquisition

Participants were imaged with a Siemens 3.0 Tesla scanner (Skyra, Siemens, Erlangen, Germany) in the Department of Radiology of the Beijing Hospital of Traditional Chinese Medicine Affiliated to Capital Medical University. Their heads were positioned within a 20 channel headcoil, and foam padding was provided to minimize head movement. During the resting-state fMRI scans, participants were required to keep awake, close their eyes, and move as little as possible. Functional data were collected by an echo planar imaging (EPI) sequence with scan parameters of repetition time (TR) = 3,000 ms, echo time (TE) = 30 ms, flip angle (FA) = 90°, field of view (FOV) = 220 × 220 mm^2^, and slice thickness = 3 mm. Sagittal structural images were acquired using a magnetization prepared rapid gradient echo (MP-RAGE) three-dimensional T1-weighted sequence (TR = 2,300 ms, TE = 2.32 ms, FA = 8°, FOV = 240 mm × 240 mm).

### Functional data preprocessing

Data analyses were performed with the Resting-State fMRI (DPARSF) toolbox (33) and SPM8 (http://www.fil.ion.ucl.ac.uk/spm/software/spm8) base on MATLAB. Firstly, the removal of the first 10 volumes, slice timing, and head-motion correction were done by preprocessing functional data. Later, the functional images were spatially normalized to the standard Montreal Neurological Institute (MNI) echo-planar imaging template and resampled to 3 × 3 × 3 mm^3^. Participants should have had no more than a maximum displacement of 3 mm in the x, y, or z axis and 3 degrees of angular motion. The regressing out of nuisance signals, including Friston 24 head motion parameters (34) and white matter and cerebrospinal fluid signals (33), was performed. Finally, linear trend and band-pass filtering (0.01–0.08 Hz) were performed to remove the influence of low-frequency drift and high-frequency noise (35).

### Voxel-wise degree centrality analysis

The resting-state fMRI degree centrality analysis “REST-DC” toolkit (REST1.8; http://www.restfmri.net) was used to calculate DC measures according to the methods used in a previous study (26). For each participant, whole-brain voxel-wise connectivity matrix was obtained by computing Pearson's correlation coefficient between the time courses of one voxel with that of every other voxel within a predefined gray matter mask. This gray matter template has been released as part of the tissue priors in SPM8 that included tissue with gray matter probabilities larger than 20%. Depending on the adjacency matrix of a graph, we calculated voxel-wise DC as in Equation (1) (26)

(1) $$\begin{array}{l} \text{DC}\left(i\right)=\sum_{j=1}^{N}r_{ij}\left(r_{ij}>r_{0}\right), \end{array}$$

where *r*_*ij*_ is the temporal Pearson's correlation of time series between voxel *i* and voxel *j*. The term *r*_0_ is a correlation threshold, which can eliminate the weak correlation (26, 36). In order to improve normality, each participants' correlation matrices were transformed into a Z-score matrix using Fisher's r-to-z transformation (37). As previously described, binary version DC was used to provide centrality characterization of functional brain networks (38). We defined FC in the whole brain between a given voxel with every other voxel based on the different correlation thresholds (*r*_0_ = 0.15, 0.2, 0.25, 0.3, and 0.35) (37, 39). The DC maps of each participant were then transformed to z-score maps in order to accord with the Gaussian distribution. The z-score transformation is achieved by subtracting the mean (DC of all voxels in brain mask) and dividing the standard deviation (DC of all voxels in brain mask). Subsequently, a 6 mm full-width-at-half-maximum (FWHM) Gaussian kernel was applied to decrease spatial noise. A two-sample *t*-test was conducted to investigate the voxel-wise DC differences in brain regions between PI patients and healthy controls in the DPARSF software. The analysis was two-tailed and was conducted after adjusting for age, gender, education level, SAS, and SDS. Multiple comparisons were corrected at the cluster-level using Gaussian random field (GRF) theory (|Z| > 1.960, cluster-wise *p* < 0.005, corrected).

According to the previous studies, five thresholds were used to compute DC in this study to avoid our primary results that were dependent on the chosen threshold (36, 40). The weighted DC was also computed, assuring the robustness of the findings with nearly identical results as shown in Figure S1.

### Seed-based FC analysis

To explore more details about resting-state FC alterations, a seed-based interregional correlation analysis was performed using the DPARSF software package. The seed region was derived from the activated brain region (PI patients' DC vs. healthy controls' DC) by creating a seeded spherical 5-mm region of interest (ROI) around the activated center of mass coordinates. Later, FC maps were generated by calculating both positive and negative correlations between the ROIs and other brain voxels. Finally, the resultant correlation maps were transformed to z-score maps using a Fisher's transformation. For each seed, group comparisons were analyzed using a two-sample *t*-test to detect voxels showing significant correlations with the seed (*p* < 0.005, GRF correction). The assessments were two-tailed and were conducted after adjusting for age, gender, education level, SAS, and SDS.

### Statistical analysis

#### Demographic analysis

Non-imaging statistical tests were performed in SPSS Statistics for Windows Version 22.0 (IBM Corporation, Armonk, NY, USA). The threshold for statistical significance was set at α < 0.05, and all hypothesis tests were two-tailed. Tests of data normality were performed using the Shapiro-Wilk test, and observations of histograms were made. Continuous variables with normal distribution were analyzed using independent *t*-tests. Otherwise, a Mann–Whitney U statistic was used to analyze the data with non-normal distribution. For categorical variables, the chi-square (χ2) test was used to compare the gender ratios.

#### Brain-behavior correlation analysis

Pearson's correlation analysis was performed to examine the association between the values of DC or FC and the clinical variables such as PSQI, SAS, SDS, and insomnia duration. Mean DC or FC values of each spherical ROI with the centroid at its corresponding peak voxel (radius = 5 mm) were extracted. The significance level was set at *P* < 0.05, for the two-tailed test.

## Results

### Clinical data

Baseline characteristics have previously been described in detail (41). A total of 26 PI patients and 28 healthy controls were enrolled. Analysis of demographic variables revealed that no group differences were detected in age, sex, and years of education (all *p* > 0.05). Significant differences were observed in PSQI, SAS, and SDS between the two groups (all *p* < 0.000, two-sample *t*-tests).

### Degree centrality analysis

**Figure 1:**
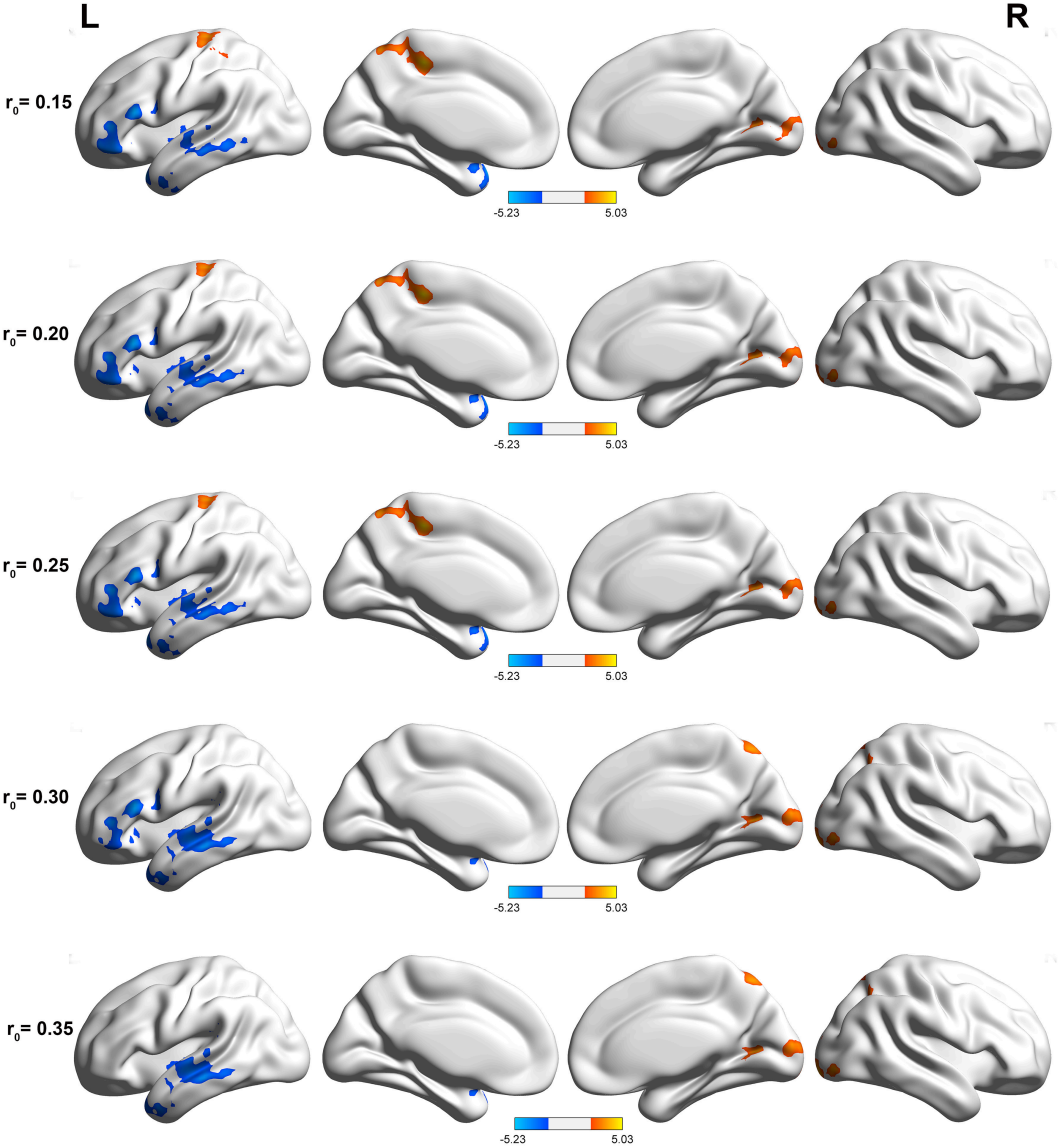
Compared with healthy controls, PI patients exhibited remarkably similar altered DC brain areas in different correlation thresholds (*r*_0_ = 0.15, 0.2, 0.25, 0.3, and 0.35). The effects are significant at a single voxel *p* < 0.05, GRF corrected cluster level *p* < 0.005. The hot (cool) color indicates significantly increased (decreased) DC in the brain area.

The DC maps of the two groups are shown in Figure 1. The results obtained from the two sample *t*-test clearly showed highly similar intragroup differences of binary DC between the two groups in several thresholds at *r*_0_ = 0.10, 0.15, 0.20, 0.25, 0.30, and 0.35. The functional hubs were mainly localized in the middle temporal gyrus (MTG), IFG, calcarine, and precuneus (Table S1). For this reason, the study mainly reported the results for DC at *r*_0_ = 0.25.

**Figure 2:**
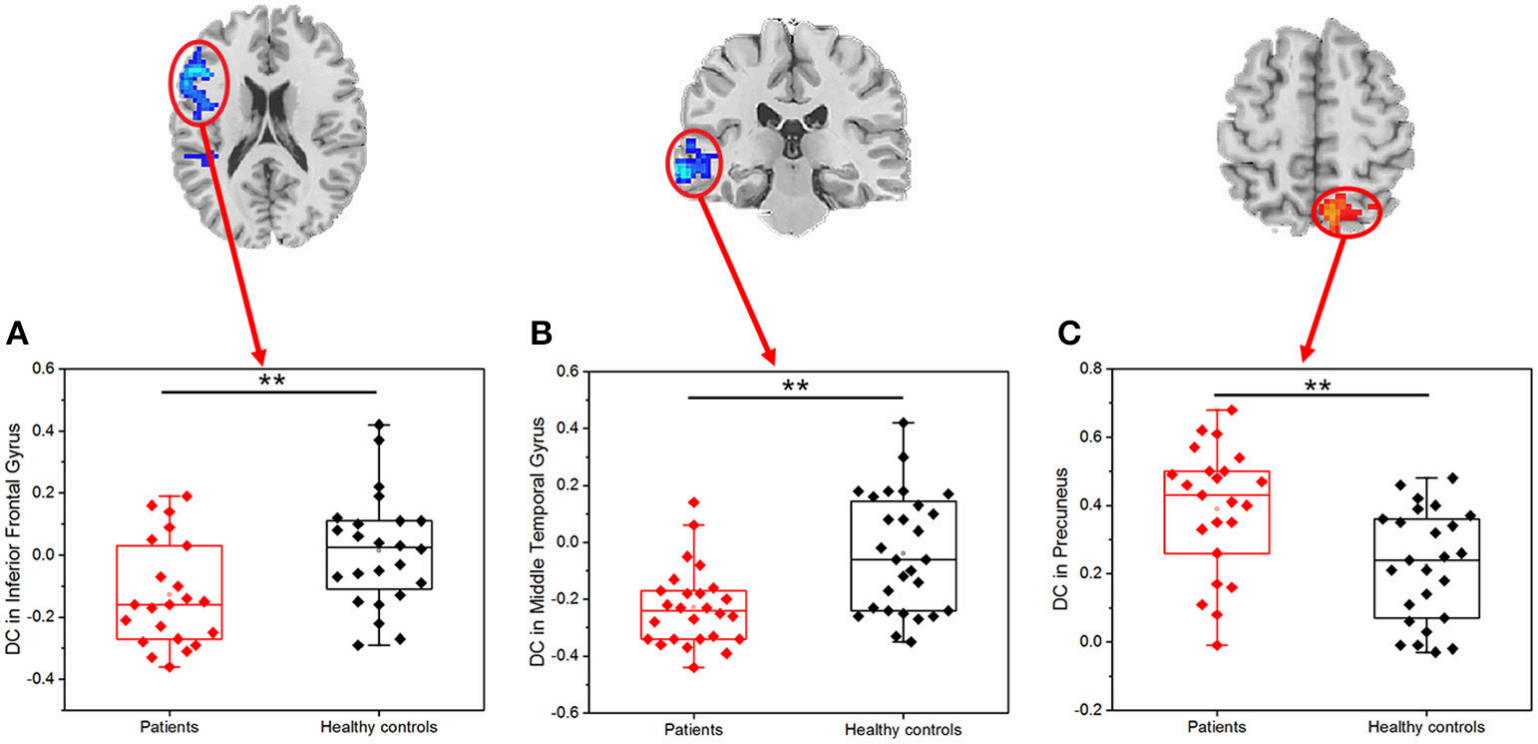
Scatter plot of DC for the significantly decreased (increased) clusters between PI patients and healthy controls (*r*_0_ = 0.25). The difference between PI patients and healthy controls based on the DC in the left inferior frontal gyrus **(A)**, left middle temporal gyrus **(B)**, and right precuneus **(C)**; Red dots: PI patients; black dots: healthy controls. Error bars represent standard deviation of the mean (**P < 0.001).

Primary insomnia patients exhibited a significantly decreased DC in the left IFG and MTG, when compared with the healthy controls (Table 1, Figure 2). These patients also demonstrated a significantly increased DC in the right precuneus region.

**Table 1:** Significant differences in degree centrality (*r*_0_ = 0.25) and functional connectivity between two groups.

	**Brain regions**	**Side**	**Condition**	**MNI coordinates**			**Cluster size**	**Peak *t*-value**
**Type**				**x**	**y**	**z**	
**DEGREE CENTRALITY**								
	Inferior frontal gyrus	Left	PI < HC	−54	24	15	540	−4.62
	Middle temporal gyrus	Left	PI < HC	−66	−27	−9	1054	−4.65
	Precuneus	Right	PI > HC	36	−63	27	763	4.08
IFG_L-seeded connectivity	No Suprathreshold clusters	–	–	–	–	–	–	–
MTG_L-seeded connectivity	Posterior Cingulate Cortex	Left	PI < HC	−3	−48	18	944	−4.35
Precuneus_R-seeded connectivity	Lateral Occipital Cortex	Right	PI > HC	42	−78	−6	633	3.24

*IGG, inferior frontal gyrus; MTG, middle temporal gyrus; L(R), left (right) hemisphere. PI, Primary insomnia; HC, healthy controls*.

### Functional connectivity analysis

**Figure 3:**
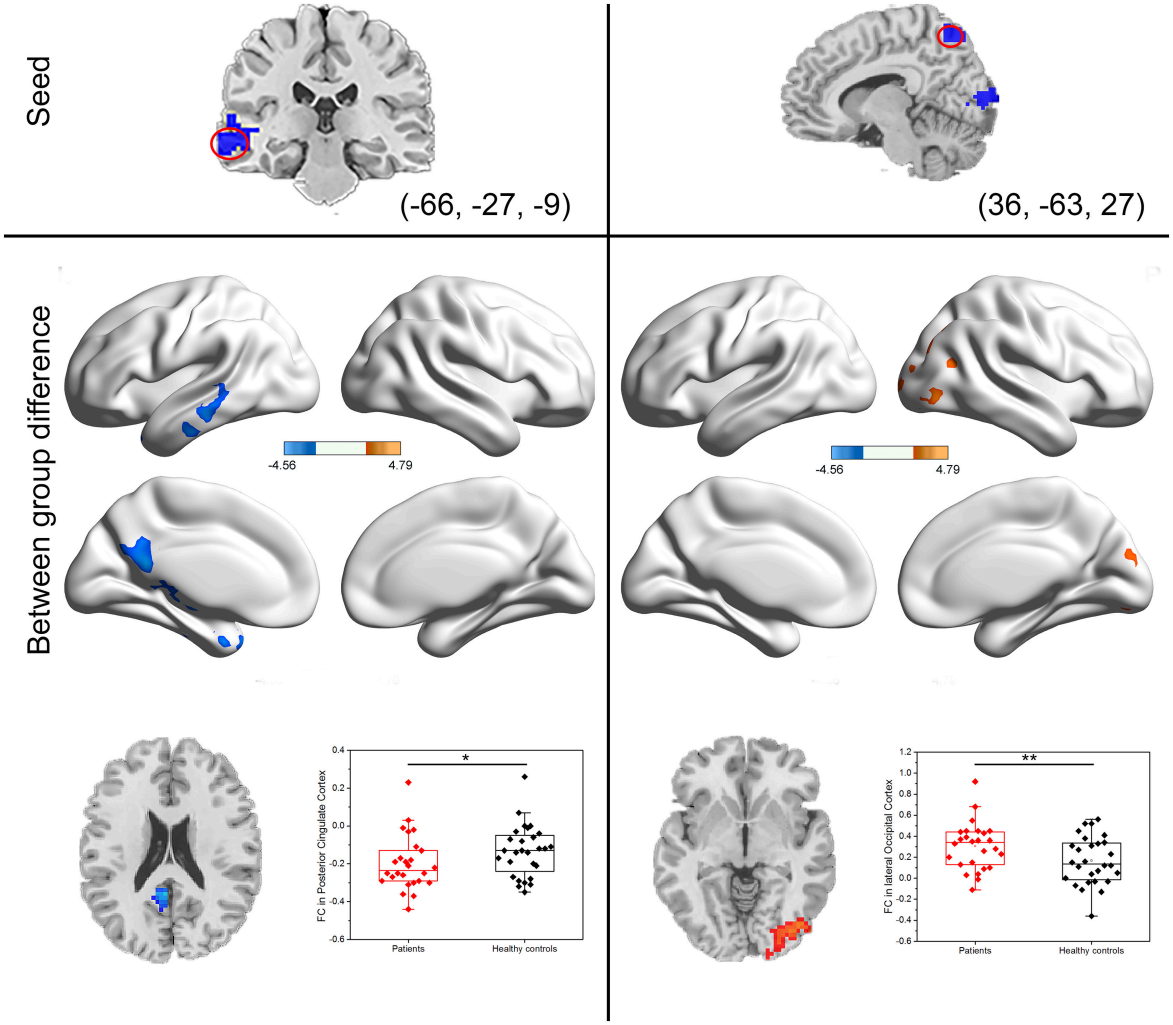
Group differences in seed-based functional connectivity. The seeds were defined as left middle temporal gyrus and right precuneus. Scatter plot of FC for the significantly increased or decreased clusters between PI patients and healthy controls. Red dots: PI patients; black dots: healthy controls. Error bars represent standard deviation of the mean (*P < 0.05, **P < 0.001).

The center points of the peak t value in brain regions (left IFG, left MTG, and right precuneus) that showed significant differences in DC between PI patients and healthy controls were defined as spherical ROIs (*r* = 5 mm). We further examined seed-based FC between the three ROIs and the whole brain regions. As compared with healthy controls, decreased FC of the left MTG found in PI patients was mainly located in the left posterior cingulate cortex (PCC) areas (Table 1, Figure 3). When compared with healthy controls, PI patients exhibited a lateralized increase in FC between the right precuneus and the right lateral occipital cortex (LOC). However, PI patients failed to reveal any suprathreshold clusters between the ROI of the left IFG and the whole brain regions.

### Correlation results

**Figure 4:**
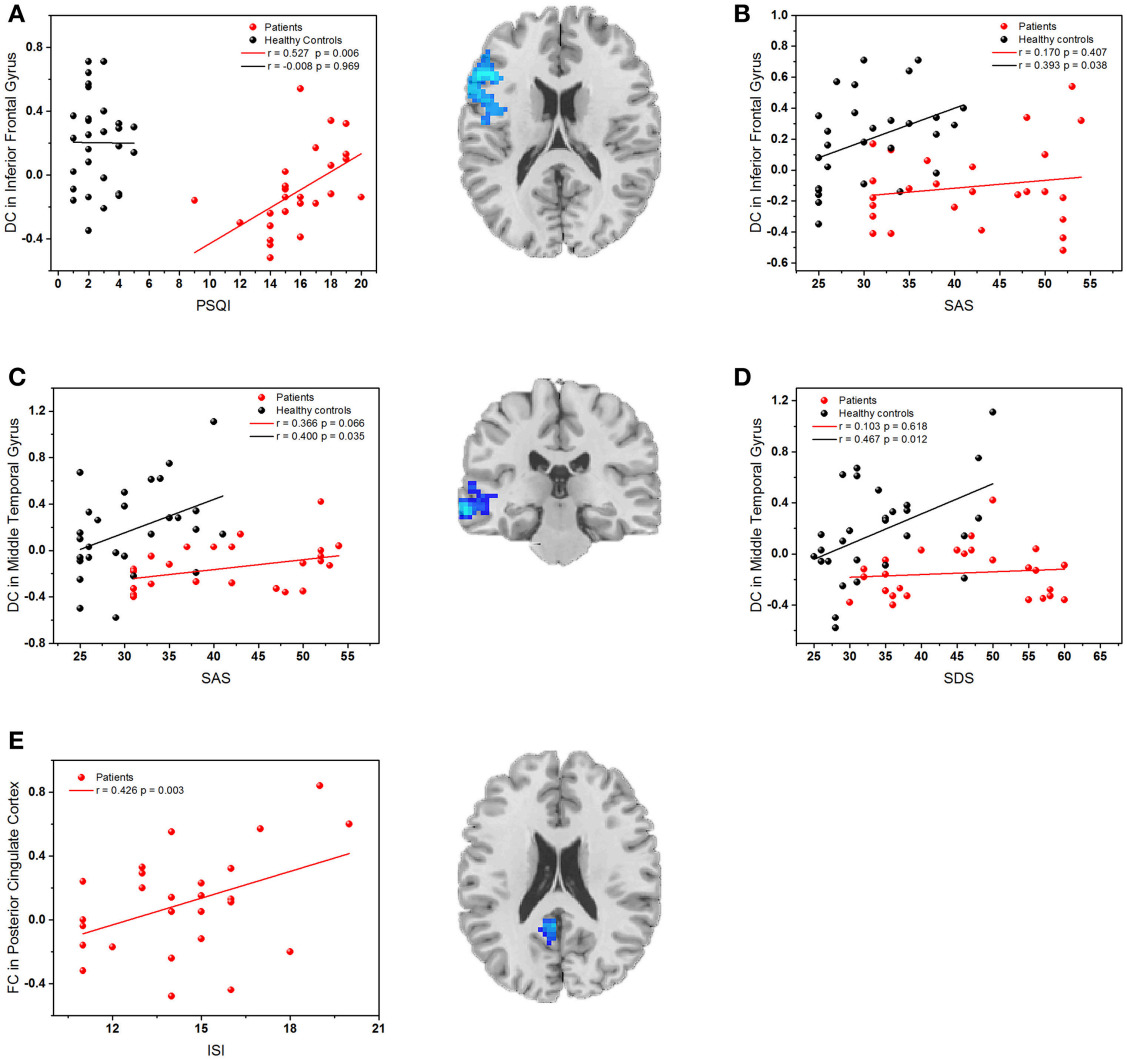
**(A)** The correlation between the PSQI scores and DC values in the left inferior frontal gyrus; **(B)** The correlation between SAS scores and DC values in the left inferior frontal gyrus; **(C)** The correlation between SAS scores and DC values in left middle temporal gyrus; **(D)** The correlation between SDS scores and DC values in left middle temporal gyrus. **(E)** The correlation between ISI scores and FC values in left posterior cingulate cortex. Red dots: PI patients; black dots: healthy controls.

The mean DC or FC values were extracted from five regions (left IFG, left MTG, and right precuneus, left PCC, and LOC) with significant group differences. As shown in Figure 4, the Pearson's correlation analyses demonstrated that the reduced DC value of the left IFG was positively correlated with PSQI (*r* = 0.527, *p* = 0.006) in PI patients, and it was also positively correlated with SAS (*r* = 0.393, *p* = 0.038) in healthy controls. Besides, the decreased DC value in the left MTG was positively correlated with SAS (*r* = 0.400, *p* = 0.035) and SDS (*r* = 0.467, *p* = 0.012) in healthy controls. Finally, the reduced FC between the left MTG and PCC was positively correlated to ISI (*r* = 0.426, *p* = 0.003). The decreased DC in the precuneus and its abnormal FC with right LOC had no relationship with any clinical variable.

## Discussion

In the current study, we investigated the abnormal intrinsic functional hubs and the functional whole-brain network in PI patients by using a combination of voxel-wise DC and seed-based FC analyses. Using a data-driven approach to investigate the degree of centrality, the results revealed that a set of cortical hubs persisted, including significantly lower DC values in the left IFG and MTG and higher DC value in the right precuneus. The seed-based FC analyses described more details about the altered functional networks anchored in these regions. Notably, these intrinsic functional hubs and the altered connectivity strength revealed linear correlation with clinical features.

Our results revealed that the IFG is one of the main cortical hubs in the brain network affected by PI. This finding is in line with the previous studies that showed the involvement of the prefrontal cortex in insomnia (13, 42–45). For example, the IFG demonstrated verbal fluency-related brain hypoactivation in chronic insomnia patients that recovered after sleep therapy (43). In addition, with the use of low frequency fluctuations (ALFF), Li et al. (45) found that PI patients showed lower ALFF value in the left IFG, and a negative correlation between the duration of PI and the ALFF value in the left IFG was observed (45). Specifically, using graph theoretical analysis, Lu et al. (25) observed that healthy participants with insomnia symptoms presented reduced regional degree and efficiency in the left IFG compared with subjects without insomnia symptoms (25). In the present study, we observed diminished DC value in the left IFG, and the DC value was positively correlated with PSQI in PI patients. Hence, our findings might speculate that IFG is a vulnerable region in the pathological process of PI. Interestingly, a positive correlation between the SAS and the DC value in the left IFG was also found in healthy controls. The prefrontal cortex has long been considered to play a vital role in emotional processing (46). Our current study provides evidence that impaired connectivity in the prefrontal cortex, specifically the IFG, might be associated with poor sleep quality under the state of insomnia, whereas the connectivity in the IFG might be associated with anxiety in healthy subjects without insomnia symptom.

The left MTG demonstrated significantly reduced DC value in PI patients in this study. The MTG is known to be a key region during both encoding and retrieval of emotional episodic memories (47, 48) and is thought to be involved in dream encoding and recall in both rapid eye movement (REM) and non-REM (NREM) sleep (49), which is also thought to be involved in the pathology of insomnia (13, 45). Using the ALFF algorithm, Li et al. (45) observed that PI patients showed higher spontaneous regional brain activity in the MTG (45). These findings suggests that insomnia is associated with altered MTG function. Structurally, PI patients had smaller volumes of gray matter in the area of MTG (13). The brain morphological results further confirmed that functional abnormalities of MTG in insomnia have an anatomical basis. In the current study, the left MTG exhibited decreased FC with the left PCC in PI patients. Previous neuroimaging studies have identified that abnormal FC in the PCC could account for insomnia disorders (50). By using positron emission tomography scans, Kay et al. (51) examined the relative regional cerebral metabolic rate for glucose (*rCMR*_*glc*_) in PI and controls with a normal sleep pattern during both morning wakefulness and NREM sleep at night, and significant group-by-state interactions in relative *rCMR*_*glc*_ were found in the PCC during non-REM sleep (51). This finding suggests that insomnia is associated with impaired disengagement of brain regions in the PCC. To supplement this, we found that reduced FC between the left MTG and the left PCC might reflect a relationship with insomnia.

The affected brain regions, the MTG and PCC, are considerably overlapped with the default-mode network (DMN), which plays an important role in consciousness modulation (52). Previous neuroimaging studies have pointed out that structural and functional abnormalities in insomnia are related to DMN alterations (19, 53–55). For instance, decreased structural connectivity between anterior and posterior regions of the DMN in the PI group has been found by using structural MRI. Moreover, decreased structural covariance within the DMN has correlation with higher PSQI scores (19). These results indicate that the disrupted DMN may implicate commonly observed sustained sleep difficulties in insomnia. Similarly, a correlation between FC values in left PCC and ISI scores was found in the current study. Our results support the fact that the functional disruption of DMN can probably be used to assess the severity of insomnia. Besides, the DC value in the left MTG was positively correlated with SAS and SDS in healthy controls. The DMN is believed to underlie self-reflective processes (56), while it is directly proportional to the subject's anxiety level when performing a task (57), and resting-state FC variations have repeatedly been verified in relation to depression (58). Our findings reveal that, in healthy subjects, higher connectivity density in the left MTG may reflect lower levels of anxiety and depression, although more studies are needed to confirm it.

The precuneus, a part of the parietal lobe, is also one of the key regions of the DMN, ascending projections to the somatosensory, cognitive, and visual cortex, and has been proven to be involved in the interwoven network of the self-conscious neural correlates during rest (59, 60). This result is consistent with selective hypometabolism in the precuneus, which has been observed in mental states of decreased or abolished consciousness, such as sleep, drug-induced anesthesia, and vegetative states (59). During wakefulness, cerebral glucose metabolism in the precuneus is at the highest level. Whereas, during slow-wave sleep and REM sleep, the precuneus is one of most deactivated brain areas (61). Furthermore, PI patients exhibited decreased spontaneous regional brain activity values in the precuneus (62). These results indicate that the abnormal precuneus functions might influence sleep quality, and our study provides the evidence of disrupted global function in the precuneus.

Moreover, an increase in FC between the right precuneus and the right LOC was observed in PI patients. Altered metabolism and spontaneous activity in the occipital cortex had been found in PI patients. The mean occipital gamma-aminobutyric acid (GABA) level was 33% lower in PI patients than in normal subjects, which was found by using single-voxel proton magnetic spectroscopy (63). Gamma-aminobutyric acid (GABA), an inhibitory neurotransmitter, has a role in the etiology and/or maintenance of insomnia (64). Decreased GABA in the occipital cortex suggests that increased activity in some neurons in the occipital cortex may result in a hyperarousal state in insomnia. Besides, an increase in parietal-occipital electroencephalographic (EEG) gamma activity was found in persons after meditative training during NREM sleep (65). Based on this finding, we might suppose that parietal-occipital EEG gamma power was a sensitive measure on brain function in sleep. We observed an increase in FC between the right precuneus and the right LOC in insomnia. The result shed new light on the pathological mechanism in insomnia.

The limitations of our study are noted below. First, as our study sample is relatively small, the results in this study need further verification in a large sample. Second, this was a cross-sectional study, and it is inadequate to identify the pattern of changes in brain activation. Thus, further longitudinal imaging studies with treatments are needed. Third, the significant association of clinical features and the pattern of changes in the brain reported in the present study should be regarded as exploratory in nature owing to the fact that no correlation persisted (*P* < 0.05) after false discovery rate (FDR) correction for multiple comparisons. Future studies with rigorous multiple testing correction need to be performed. Fourth, fMRI data from wakefulness and a deep sleep state are essential for us to understand how brain function is influenced by insomnia. A major methodological constraint is that sleep in a scanner will be difficult to achieve, therefore, only waking state fMRI data were obtained in this study. Besides, the affected areas in PI patients in our study are diffuse, and it is difficult to establish their participation in the insomnia disorder. However, a meta-analysis clearly indicated that a wide range of brain alterations was presented in insomnia disorder (66). Further studies on the pathogenic mechanism of insomnia are needed to reveal the specific role of these affected brain regions in insomnia disorder.

In summary, this research used voxel-wise DC and seed-based FC to investigate the intrinsic functional hubs or whole brain functional connection changes in PI patients. The results revealed that PI patients exhibited lower DC values in the left LFG and MTG and higher DC values in the right precuneus. Decreased FC strength was observed between the left MTG and the left PCC, and increased FC was also found between the right precuneus and the right LOC. Furthermore, reduced DC in left the LFG and decreased FC in the left PCC were positively correlated with sleep quality. The findings of this study provide a better understanding of the nature of disconnection in PI patients, which may be helpful to figure out the neurobiological mechanism of insomnia.

## Ethics statement

The Research Ethical Committee of Beijing Hospital of Traditional Chinese Medicine Affiliated to Capital Medical University approved the trial.

## Author contributions

Q-QL conceived and designed the experiments. J-WH, PZ, Q-NF, JZ, and Z-YW performed the experiments. XW, X-RW, and J-LL analyzed the data. C-QY, XW, and C-ZL wrote the paper. All authors approved the final manuscript.

### Conflict of interest statement

The authors declare that the research was conducted in the absence of any commercial or financial relationships that could be construed as a potential conflict of interest.

## Footnotes

^1^Aaos Medicine. *International Classification of Sleep Disorders*. 3*rd Ed. Darien, IaaOSM*.

^2^DSM-IV. The Fourth Edition of the Diagnostic and Statistical Manual of Mental Disorders.
