# Supplementary material for: Abnormal Global Brain Functional Connectivity in Primary Insomnia Patients: A Resting-State Functional MRI Study
Source: Front Neurol. 2018 Nov 2;9:856. doi: 10.3389/fneur.2018.00856 (PMC6224336; doi:10.3389/fneur.2018.00856)
Supplement: Supplementary file 1 [file Table_1.DOCX]

**Supplementary Information**

**Abnormal global brain functional connectivity in primary insomnia patients: a resting-state functional MRI study**

Chao-Qun Yan^1.2#^; Xu Wang^3#^; Jian-Wei Huo^4^;Ping Zhou^2^; Jin-ling Li^1^; Zhong-Yan Wang^4^; Jie Zhang^2^; Qing-Nan Fu^2^; Xue-Rui Wang^2^; Cun-Zhi Liu^1*^; Qing-Quan Liu^2*^

1. Department of Acupuncture and Moxibustion, Dongfang Hospital, Beijing University of Chinese Medicine. No. 6 Fangxingyuan, 1st Block, Fengtai District, Beijing, 100078, China.
2. Department of Psychosomatic Medicine, Beijing Hospital of Traditional Chinese Medicine affiliated to Capital Medical University, 23 Meishuguanhou Street, Dongcheng District, Beijing 100010, China.
3. School of Life Sciences, Beijing University of Chinese Medicine, 11 Beisanhuan East Road, Chaoyang District, Beijing 100029, China.
4. Department of Radiology, Beijing Hospital of Traditional Chinese Medicine affiliated to Capital Medical University, 23 Meishuguanhou Street, Dongcheng District, Beijing 100010, China

# These authors contributed equally to this study.

*Corresponding author at:

Qing-Quan Liu

Beijing Hospital of Traditional Chinese Medicine Affiliated to Capital Medical University, 23 Meishuguanhou Street, Dongcheng District, Beijing 100010, China. E-mail address: [liuqingquan2003@126.com](mailto:liuqingquan2003@126.com);

Cun-Zhi Liu

Department of Acupuncture and Moxibustion, Dongfang Hospital, Beijing University of Chinese Medicine. No. 6 Fangxingyuan, 1st Block, Fengtai District, Beijing, 100078, China. E-mail address: lcz623780@126.com.


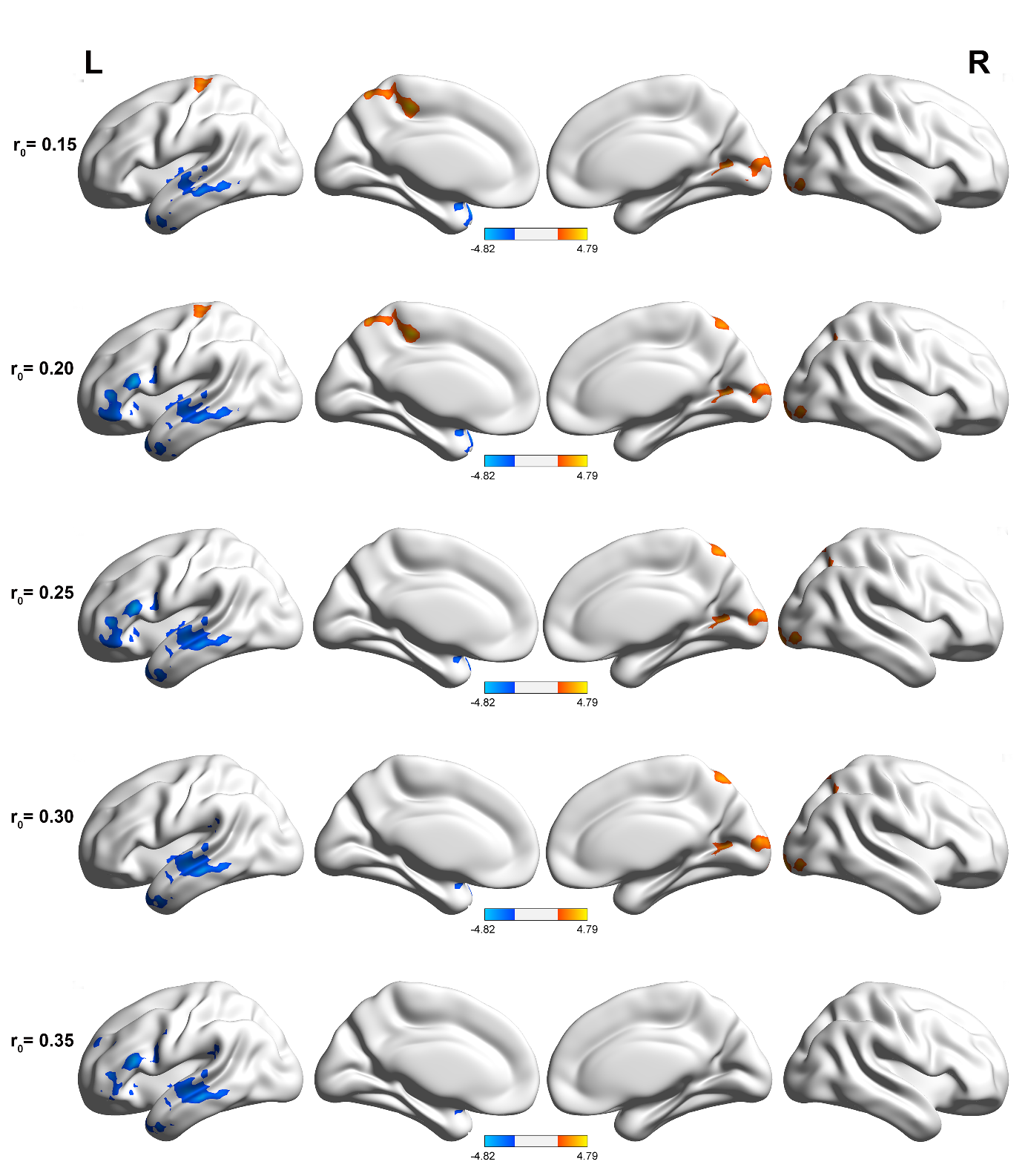


**Figure S1.** Weighted DC map. The PI patients showed remarkably similar altered DC brain areas according to different correlation thresholds when compared to healthy controls (r_0_ = 0.15, 0.2, 0.25, 0.3 and 0.35). The effects are significant at single voxel p < 0.05, GRF corrected cluster level p < 0.005. The hot (cool) color indicates significantly increased (decreased) DC brain area.

Table 1 Significant differences in degree centrality and functional connectivity between two groups.

|  | **Brain regions** | **Side** | **Condition** | **MNI coordinates** | | | **Cluster size** | **Peak t value** |
| --- | --- | --- | --- | --- | --- | --- | --- | --- |
|  |  |  |  | **x** | **y** | **z** |  |  |
| **Degree Centrality** |  |  |  |  |  |  |  |  |
| r_0_ = 0.15 | Middle Temporal Gyrus | Left | PI < HC | -42 | 15 | -27 | 841 | -5.23 |
|  | Inferior Frontal Gyrus | Left | PI < HC | -54 | 24 | 15 | 464 | -4.55 |
|  | Calcarine | Right | PI > HC | 9 | -72 | -18 | 471 | 4.51 |
|  | Precuneus | Left | PI > HC | -9 | -30 | 51 | 651 | 4.58 |
| r_0_ = 0.20 | Middle Temporal Gyrus | Left | PI < HC | -42 | 15 | -27 | 1010 | -4.82 |
|  | Inferior Frontal Gyrus | Left | PI < HC | -54 | 24 | 15 | 512 | -4.66 |
|  | Calcarine | Right | PI > HC | 9 | -72 | -18 | 540 | 4.57 |
|  | Precuneus | Left | PI > HC | -9 | -30 | 51 | 543 | 4.61 |
| r_0_ = 0.30 | Middle Temporal Gyrus | Left | PI < HC | -66 | -27 | -9 | 999 | -4.76 |
|  | Inferior Frontal Gyrus | Left | PI < HC | -54 | 24 | 15 | 532 | -4.48 |
|  | Precuneus | Right | PI > HC | 36 | -63 | 27 | 781 | 4.28 |
| r_0_ = 0.35 | Middle Temporal Gyrus | Left | PI < HC | -66 | -27 | -9 | 929 | -4.77 |
|  | Inferior Frontal Gyrus | Left | PI < HC | -48 | 24 | 18 | 659 | -4.40 |
|  | Precuneus | Right | PI > HC | 36 | -63 | 27 | 792 | 4.33 |

Abbreviation: MNI, Montreal Neurological Institute.

**Response to Reviewers**

1. **Response to Reviewer 1**

**Q 1 Please summarize the main findings of the study.**

**Response:** We thank the reviewer for the comments.

**Q 2 Please highlight the limitations and strengths of the study.**

**2.1 The study shows findings from a high number of cases.**

**Response:** We are very grateful for the reviewers’ acknowledgement of our efforts.

**2.2 The affected areas are diffuse and thus it is difficult to establish their participation in the sleep generation.**

**Response:** We are very grateful for the reviewer’s valuable suggestion. As mentioned by the reviewer, the affected areas that found in our study are diffuse. We agree with the reviewer that it is difficult to establish their participation in the sleep generation. We have added this limitation in the Discussion section. The main changes have been highlighted **in red** in the highlighted of manuscript.

However, insomnia is associated with complex pathophysiological changes in automonic and central nervous system. The hyperarousal is a key component of the pathophysiology in insomnia ^[1]^. It implicates an imbalance of sleep–wake regulation, which consists of either overactivity of the arousal systems, hypoactivity of the sleep-inducing systems, or both ^[2]^. Any brain areas relevant for sleep regulation might also be capable of inducing insomnia. What’s more, a meta-analysis demonstrated that the affected areas were diffuse in patients with insomnia disorder compared with healthy subjects (Appendix Figure 1) ^[3]^.

The pathological changes of insomnia disorder are diversity and complexity, and our findings are only the tip of the iceberg. The affected areas in PI patients were found in our study are diffuse, and it may difficult to establish their specific participation in the insomnia disorder. With more and more studies on pathogenic mechanism of insomnia, researches may reveal those key role brain regions in insomnia disorder.

References

1. Kalmbach DA, Cuamatzi-Castelan AS, Tonnu CV, et al. Hyperarousal and sleep reactivity in insomnia: current insights. Nature and science of sleep 2018;10:193-201 doi: 10.2147/NSS.S138823.
2. Riemann D, Nissen C, Palagini L, et al. The neurobiology, investigation, and treatment of chronic insomnia. Lancet Neurol 2015;14(5):547-58 doi: 10.1016/S1474-4422(15)00021-6.
3. Tahmasian M, Noori K, Samea F, et al. A lack of consistent brain alterations in insomnia disorder: An activation likelihood estimation meta-analysis. Sleep Med Rev 2018 doi: 10.1016/j.smrv.2018.07.004.


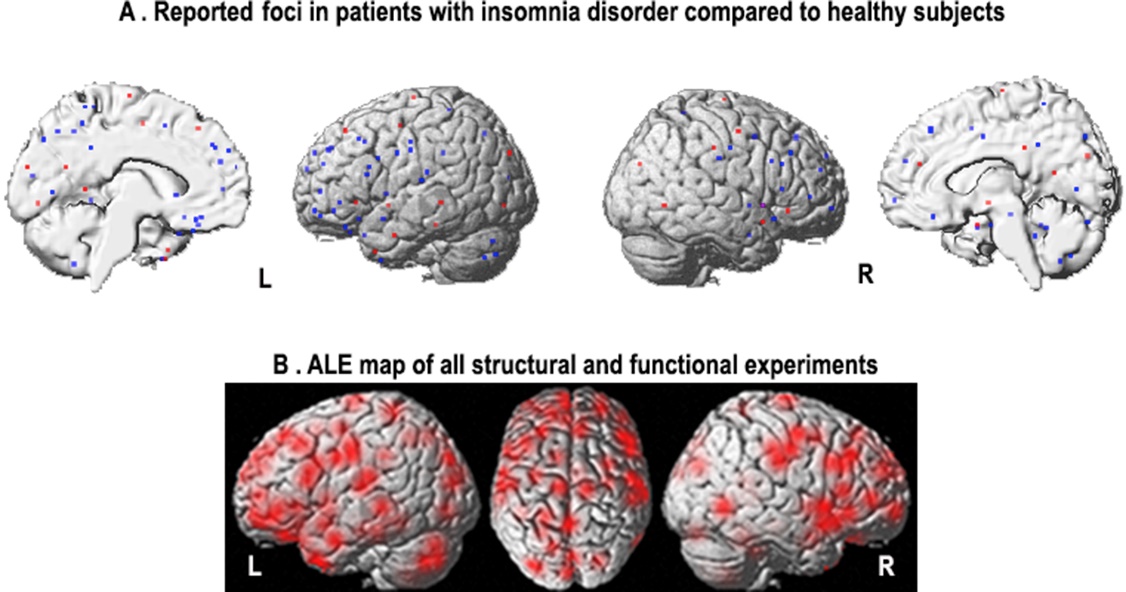


Appendix Figure 1. A) Reported coordinates reflecting structural/functional brain alterations in patients with insomnia disorder compared to healthy subjects (red = increased, blue = decreased); B) Activation likelihood estimation (ALE) maps relevant to meta-analysis, reflecting spatial uncertainty associated with each peak coordinate by modeling Gaussian probability distributions around each peak coordinate (p = 0.914, family-wise error in cluster level). [3]

**Q 3 Are there objective errors in the methods or results, and are the conclusions supported by the presented data?**

**Conclusions are supported by the results but they are vague because differences were observed in diffuse cortical areas.**

**Response:** We are very grateful for the reviewer’s constructive suggestion. For detail, please see the response of **Q 2**.

**Q 4 Check List**

**Response:** We thank the reviewer for the positive feedback to our work. The language in the manuscript has been improved by professional native English editing.

**Q 5 Please provide your detailed review report to the authors:**

- 1. **The sentence in page 12: "Our findings revealed that higher connectivity density in the left MTG, and the lower anxiety and depression mood in the healthy subjects without insomnia, although more studies are needed to confirm it" I do not understand the relation between connectivity density and anxiety. Please, explain in detail or remove the sentence.**

**Response:** We are very grateful for the reviewer’s constructive suggestion. We are sorry for the unclear description in the manuscript. We have modified this sentence in the Discussion section, as shown below.

Our findings revealed that, in healthy subjects, higher connectivity density in the left MTG may reflect lower levels of anxiety and depression, although more studies are needed to confirm it.

- 1. **A reduction of the gray matter volume may be detected in the fMRI?**

**Response:** We are very grateful for the insightful suggestion. We are terribly sorry for the confusion. We have deleted the wrong place. The studies, cited in the manuscript, about gray matter volume clearly stated that it was detected by structural MRI. If there still has some confusion, please let us know.

- 1. **Authors indicate that there is a reduction of GABA activity in the occipital lobe. Is it specific in this region? Why?**

**Response:** We thank the reviewer’s for the insightful comments. The role of g-aminobutyric acid (GABA) in sleep induction and maintenance has been well received after most insomnia treatments targeting GABAa receptors ^[1]^. Using single-voxel proton MRS (1H-MRS), a study averaging GABA content from several brain regions (i.e., not specific in occipital cortex) found that GABA levels were reduced by approximately 30% in primary insomnia subjects ^[2]^. A replication study by Plante et al. in a different cohort of PI subjects, showed that brain GABA reduction in PI in the occipital cortex and anterior cingulate cortex were 33% and 21%, respectively ^[3]^. According to these studies, we can draw a conclusion that the reduction of GABA activity in the occipital cortex is not a specific region for primary insomnia, but is the most obvious region.

References

1. Avidan AY, Neubauer DN. Chronic Insomnia Disorder. Continuum (Minneap Minn) 2017;23(4, Sleep Neurology):1064-92 doi: 10.1212/01.CON.0000522244.13784.bf.
2. Winkelman JW, Buxton OM, Jensen JE, et al. Reduced brain GABA in primary insomnia: preliminary data from 4T proton magnetic resonance spectroscopy (1H-MRS). Sleep 2008;31(11):1499-506.
3. Plante DT, Jensen JE, Schoerning L, et al. Reduced gamma-aminobutyric acid in occipital and anterior cingulate cortices in primary insomnia: a link to major depressive disorder? Neuropsychopharmacology 2012;37(6):1548-57 doi: 10.1038/npp.2012.4.
   1. **Authors should check references because sometimes appear with the complete name of the Journal and sometimes with the abbreviation.**

**Response:** Thanks very much for the reviewer’s valuable opinion. We have checked the references, and amend all references according to the reference preparation guidelines of the journal.

**Q 12 What is the level of revision required based on your comments: Minor revisions**

**Response:** We are very grateful for your acknowledgement of our efforts. We have carefully revised the manuscript according to your construct suggestion. We hope that the reviewer will find this version suitable for publication in ***Frontiers in Neurology.***

1. **Response to Reviewer 2**

**Q 1 Please summarize the main findings of the study.**

**Response:** We are very grateful for your comments.

**Q 2 Please highlight the limitations and strengths of the study.**

- 1. **The results needs verification in a large sample.**

**Response:** We thank the reviewer for the kind suggestion. We agree with the reviewer that our study sample is relatively small. The results of this study need further verification in a large sample. We have added this limitation in the Discussion section.

- 1. **Limitations are the same that are presented in other studies, per example, related to the unlikely possibility that patients achieve enough sleep in a scanner.**

**Response:** We are very grateful for your constructive suggestion. As mentioned by the reviewer, it is useful to collect fMRI data both in wakefulness and sleep states. The pathological mechanisms of insomnia will be better understood by combing those data. However, a major methodological constraint is that sleep in a scanner will be difficult to achieve, therefore only waking state fMRI data was obtained in this study. We have added this limitation in the Discussion section.

- 1. **This study showed abnormal intrinsic functional hubs in insomnia patients using a voxel-wise degree centrality (DC) analysis and seed-based functional connectivity (FC) approach. The findings of this study showed a reduction of connectivity between differents areas with higher DC values on right precuneus that suggests abnormal mechanisms useful to subtyping insomnias.**

**Response:** We thank the reviewer for the comments.

**Q 3 Are there objective errors in the methods or results, and are the conclusions supported by the presented data?**

**The conclusions are supported by the presented data.**

**I didnt find errors in Methods or Results**

**Response:** Thank very much for the positive feedback about our work.

**Q 4 Check List**

**Response:** We are very grateful for your acknowledgement of our efforts.

**Q 5 Please provide your detailed review report to the authors.**

**This study demonstrate that insomniac patients exhibit abnormal intrinsic connections in temporal and parietal (right precuneus) lobes and the default-mode network. These findings contribute to understand the pathophysiology of chronic insomniacs. This study showed abnormal intrinsic functional hubs in PI patients using a voxel-wise degree centrality (DC) analysis and seed-based functional connectivity (FC) approach. The findings of this study showed a reduction of connectivity between differents areas with higher DC values on right precuneus that suggest abnormal mechanisms useful to subtyping insomnias.**

**The results needs verification in a large sample.**

**Limitations are the same that are presented in other studies,per example, related**

**to unlikely possibility that patients achieve enough sleep in a scanner.**

**Response:** Thanks very much for the reviewer’s insightful comments***.*** For more detail about the response, please see the responses of Q2.

**Q 12 What is the level of revision required based on your comments: No answer given.**

**Response:** We thank to the reviewer for the very much for the positive feedback to our work.

**Highlight of Manuscript**

**Abstract**

**Background:** Resting-state functional magnetic resonance imaging (fMRI) studies have uncovered the disruptions of functional brain networks in primary insomnia (PI) patients. However, the etiology and pathogenesis underlying this disorder remains ambiguous, and the insomnia related symptoms are inﬂuenced by a complex network organization in the brain. The purpose of this study was to explore the abnormal intrinsic functional hubs in PI patients using a voxel-wise degree centrality (DC) analysis and seed-based functional connectivity (FC) approach.

**Methods:** Twenty-six PI patients and 28 healthy controls were enrolled and underwent resting-state fMRI. DC was measured across the whole brain, and group differences in DC were compared. The peak points, which significantly altered DC between the two groups, were defined as the seed regions and further used to calculate FC of the whole brain. Then, correlation analyses were performed between the changes in brain function and clinical features.

**Results:** PI patients showed lower DC values than healthy controls in the left inferior frontal gyrus (IFG) and middle temporal gyrus (MTG), and higher DC value in the right precuneus. The seed-based analyses demonstrated decreased FC between the left MTG and the left posterior cingulate cortex (PCC), and decreased FC between the right precuneus and the right lateral occipital cortex. Reduced DC in the left IFG and decreased FC in the left PCC were positively correlated with Pittsburgh sleep quality index and Insomnia Severity Index.

**Conclusions:** This study revealed that PI patients exhibited abnormal intrinsic functional hubs in the left IFG, MTG and the right precuneus, as well as abnormal seed-based FC in these hubs. The results contribute to better understand how brain function inﬂuences the symptoms of PI.

**Keywords:** Primary insomnia; Sleep disorder, Degree Centrality; Functional connectivity; Resting-state fMRI

**1. Introduction**

The central feature of primary insomnia (PI) is dissatisfaction with sleep quantity or quality, associated with difficulty falling asleep, maintaining sleep, or early morning awakening (Buysse et al., 2017). Insomnia is a very common health problem that affects 30% to 35% of adults on an episodic basis and 10% to 12% on a chronic basis (AAoS Medicine. International classification of sleep disorders. 3rd ed. Darien;Ohayon, 2002). One third or more of the population suffers daily from a sleep disturbance or excessive daytime sleepiness (Partinen, 2011). Insomnia is associated with an increased risk of Alzheimer's disease (Peter-Derex et al., 2015), Parkinson's disease (Gan-Or et al., 2018), hypertension (Thomas and Calhoun, 2017), cardiovascular diseases (Li et al., 2014a), depression (Kilic et al., 2017), obesity (Chan et al., 2017), type 2 diabetes (Tan et al., 2017), and mortality (Akerstedt et al., 2017). Not surprisingly, insomnia has significant economic and societal impacts. It causes an equivalent to annualized population-level estimates of 63.2 billion reduction of the total America workforce due to lost work performance or absenteeism (Kessler et al., 2011). It is crucial to understand the pathologic changes in insomnia by exploring the central nervous system. Findings could offer novel interventional treatments for those patients. However, the etiology and pathogenesis underlying this disorder remains uncertain.

Brain imaging technology has proven to be informative for investigating the central mechanism of PI. Structural neuroimaging studies have revealed brain tissue injury associated with PI by using voxel-based morphometry (VBM) and diffusion tensor imaging analyses (Joo et al., 2013;Li et al., 2016c). Specifically, the abnormal regional gray matter volume or white matter integrity have been shown in multiple brain regions, including the anterior cingulate cortex (Winkelman et al., 2013), hippocampal (Joo et al., 2014), medial frontal and middle temporal gyri (Joo et al., 2013), thalamus, internal capsule, anterior corona radiate, and corpus callosum (Li et al., 2016c). The structural changes are usually accompanied by an impairment of brain function. Some studies have reported the abnormal spontaneous functional activity in PI patients by using low frequency fluctuations (Liu et al., 2016), regional homogeneity (Wang et al., 2016), and seed-based functional connectivity (FC) approach (Nie et al., 2015). Although abnormal structural and functional properties in many brain regions have been found in PI patients, these observations did not provide information about integrated global brain function alterations. However, the human brain is a complex and well organized with coordination of different brain regions as a functional network (Lehrer, 2009;Xia and He, 2017). It is necessary to investigate the brain connectivity within whole-brain network.

Recently, modern developments in graph theory have delivered important insights into functional brain networks (Strogatz, 2001). Graph theoretical analysis is a large-scale method, and has become an increasingly useful tool to explore the systematical alteration of whole-brain functional organization and connection (Bullmore and Sporns, 2009). A graph theoretical analysis based on an automated anatomical labeling atlas study found that chronic insomnia patients showed altered topological characteristics of functional brain networks, expressed as altered nodal in the default mode network, dorsal attention network, and sensory-motor network regions (Li et al., 2018b). Using graph theoretical analysis based on defined nodes and edges of the networks previously, healthy subjects with insomnia symptoms demonstrated reduced regional degree and efficiency in the left inferior frontal gyrus compared with healthy subjects without insomnia symptoms (Lu et al., 2017). Nevertheless, the altered topological properties of the global functional brain network at the voxel level in PI patients are not clear.

Voxel-wise degree centrality (DC) is a graph theory-based and data driven approach, which can evaluate the importance of each voxel in the brain, and represents its connectivity strength to every voxel (Zuo et al., 2012). The index of DC is a better connectivity metric than other measurements, because it can assign a higher value to a voxel when this voxel has stronger connections with other voxels in the brain network. DC emphasizes the impact and significance of a network at voxel level, and reflects the functional brain network “hub” properties in the network information communication (Telesford et al., 2011;Tomasi and Volkow, 2011). In a functional brain network, hub regions play pivotal roles in the coordination of information flow (Sporns et al., 2007), and are consistent and stable in healthy human brain, but highly vulnerable to pathological processes (Cole et al., 2013;Zanto and Gazzaley, 2013). DC measures based on resting-state functional magnetic resonance imaging (fMRI) has been used to observe the alterations of functional networks in diverse diseases, and exhibits relatively high test–retest reliability (Zuo and Xing, 2014). Focused on the voxel-wise DC may provide a novel insight into the pathogenesis of the PI.

In the present study, DC between the PI patients and healthy controls were compared to identify significant alterations in intrinsic functional hubs. Then, we further performed seed-based FC analyses, using the seed regions with significant alterations in the DC analysis, for better detecting detailed information regarding the connectivity in those hubs. Next, we evaluated the relationships between the clinical features and the DC or FC values. The current study will contribute to the further understanding of the mechanism of PI, and provide a bridge for future studies.

**2. Materials and method**

2.1 Participants

Participants were enrolled from 2009 September 2014 through September 2016 at the Beijing Hospital of Traditional Chinese Medicine Affiliated to Capital Medical University. The cohort included 30 patients with PI, and 30 healthy controls without insomnia symptoms. Of note, PI patients were recruited in outpatient clinics from the Department of Psychosomatic Medicine, while healthy controls were recruited primarily through advertisements in the community

The inclusion criteria for PI patients were as follows: (i) age from 25 to 60 years, (ii) right-hand dominance, (iii) meeting the DSM-IV inclusion criteria for PI, (iv) reporting difficulty in falling asleep, maintaining sleep, or early awakening at the same time for at least 1 month, (v) absence of psychoactive medication use at least 2 weeks before and during the study. PI Patients were excluded if they had any of the following: (i) other sleep disorders (e.g., hypersomnia or parasomnia), (ii) insomnia associated with specific reasons such as drugs, alcohol, or physical and mental illness, (iii) history of heart disease, stroke, nephritis or psychiatric diseases, (iv) abnormalities in brain structure such as tumors or subdural hematomas.

The healthy controls were age-, sex-, hand dominance-, and education-level-matched to PI patients, and were included if they met the following criteria: (i) good sleep quality and a Pittsburgh sleep quality index score < 3, (ii) regular sleep habits, (iii) absence of significant heart, lung disease, neurological or major psychiatric disorder, (iv) normal conventional brain magnetic resonance imaging (MRI).

Demographics of the PI patients and HC, including age, sex, and education years, were collected. Self-rating Anxiety Scale (SAS), the Self-rating Depression Scale (SDS), Pittsburgh sleep quality index (PSQI), and Insomnia Severity Index (ISI) were also measured. All participants underwent MRI scan. Four PI patients and two healthy controls were excluded before data analysis due to incomplete MRI data or excessive head motion.

2.2 Standard protocol approvals, registrations, and patient consents.

All study procedures were approved by the Research Ethical Committee of Beijing Hospital of Traditional Chinese Medicine Affiliated to Capital Medical University (reference: 2014BL-003-01). Written informed consent was obtained from participants. All experiments were performed in accordance with relevant guidelines and regulations.

2.3 MRI acquisition

Participants were imaged with a Siemens 3.0 Tesla scanner (Skyra, Siemens, Erlangen, Germany) in the Department of Radiology for Beijing Hospital of Traditional Chinese Medicine Affiliated to Capital Medical University. Their head were positioned within an eight-channel headcoil, and foam padding was provided to minimize head movement. During the resting-state fMRI scans, participants were required to keep awake, close their eyes, and move as little as possible. Functional data were performed by an echo planar imaging (EPI) sequence with scan parameters of repetition time (TR) = 3000 ms, echo time (TE) = 30 ms, flip angle (FA) = 90°, field of view (FOV) =220×220 mm^2^, and slice thickness = 3 mm. Sagittal structural images with a were acquired using a magnetization prepared rapid gradient echo (MP-RAGE) three-dimensional T1-weighted sequence (TR = 2300 ms, TE = 2.32 ms, FA = 8°, FOV = 240 mm × 240 mm).

2.4 Functional data preprocessing

Data analyses were performed with the Resting-State fMRI (DPARSF) toolbox (Chao-Gan and Yu-Feng, 2010) and SPM8 (http://www.fil.ion.ucl.ac.uk/spm/software/spm8) for MATLAB. Firstly, the removal of first 10 volumes, slice timing, and head-motion correction were done in functional data preprocessing data. Then, the functional images were spatial normalized to the standard Montreal Neurological Institute (MNI) echo-planar imaging template and resampled to 3 × 3 × 3 mm^3^. Participants should have no more than 3 mm maximum displacement in x, y, or z and 3 degrees of angular motion. Regressing out nuisance signals was performed including Friston 24 head motion parameters (Satterthwaite et al., 2013), white matter and cerebrospinal fluid signals (Chao-Gan and Yu-Feng, 2010). Finally, the linear trend and band-pass filtering (0.01–0.08Hz) were performed to remove the influence of low-frequency drift and high-frequency noise (Cordes et al., 2001).

2.5 Voxel-wise degree centrality analysis

The resting-state fMRI degree centrality analysis “REST-DC” toolkit (REST1.8; http://www.restfmri.net) was used to calculate DC measures according the methods used in previous study (Zuo et al., 2012). For each participant, whole-brain voxel-wise connectivity matrix was obtained by computing Pearson’s correlation coefficient between the time courses of one voxel with that of every other voxel within a predefined grey matter mask. This grey matter template has been released as a part of the tissue priors in SPM8 that included tissue with grey matter probabilities larger than 20%. Depending on the adjacency matrix of a graph, we calculated voxel-wise DC as in equation (1) (Zuo et al., 2012)

$\mathrm{DC}\left( i \right)=\sum_{j=1}^{N} r_{ij}(r_{ij}>r_{0})$ (1)

where $r_{ij}$ is the temporal Pearson’s correlation of time series between voxel $i$ and voxel $j$. The r_0_ is a correlation threshold, which can eliminate the weak correlation (Zuo et al., 2012;Li et al., 2016b). In order to improve normality, each participant correlation matrices were transformed into a Z-score matrix using Fisher’s r-to-z transformation (Takeuchi et al., 2015). As previously described, binary version DC were used to provide centrality characterization of functional brain networks (Chen et al., 2016). We defined functional connectivity in the whole brain between a given voxel with every other voxel based on the different correlation thresholds (r_0_ = 0.15, 0.2, 0.25, 0.3 and 0.35) (Takeuchi et al., 2015;Luo et al., 2017). The DC maps of each participant were then transformed to z-score maps in order to accord with the Gaussian distribution. The z-score transformation is achieved by subtracting mean (DC of all voxels in brain mask) and dividing standard deviation (DC of all voxels in brain mask). Subsequently, a 6 mm full-width-at-half-maximum (FWHM) Gaussian kernel was applied to decrease spatial noise. A two-sample t-test was conducted to investigate the voxel-wise DC differences in brain regions between HC and healthy controls in the DPARSF software. The analysis were two-tailed adjusting for age, gender, education level, SAS, and SDS. Multiple comparisons were corrected at the cluster-level using Gaussian random field (GRF) theory (|Z| > 1.960, cluster-wise p < 0.005, corrected).

According to the previous studies, five thresholds were used to compute DC in this study to avoid our primary results were dependent on the chosen threshold (Li et al., 2016b;Xiao et al., 2016). The weighted DC was also computed, assuring the robustness of the findings with nearly identical results as shown in Supplementary Figure S1.

2.6 Seed-based FC analysis

To explore more details about resting-state functional connectivity alterations, a seed-based interregional correlation analysis was performed using the DPARSF software package. The seed region was derived from the activated brain region (PI patients DC vs. healthy controls DC) by creating a seeded spherical 5-mm region of interest (ROI) around the activated center of mass coordinates. Then, functional connectivity maps were generated by calculating both positive and negative correlations between the ROIs and other brain voxels. Finally, the resultant correlation maps were transformed to z-score maps using a Fisher’s transformation. For each seed, group comparisons were analyzed using two-sample t-test to detect voxels showing significant correlations with the seed (p < 0.005, GRF correction). The assessments were two-tailed adjusting for age, gender, education level, SAS, and SDS.

2.7 Statistical analysis

Demographic analysis

Non-imaging statistical tests were performed in SPSS Statistics for Windows Version 22.0 (IBM Corporation, Armonk, NY, USA). The threshold for statistical significance was set at α < 0.05, and all hypothesis tests were two-tailed. Tests of data normality were performed using the Shapiro-Wilk Test and observation of histograms. Continuous variables with normal distribution were analyzed using independent t-tests. Otherwise, a Mann–Whitney U statistic was used to analyze the data with non-normal distribution. For categorical variables, the chi-square (χ2) test was used to compare the gender ratios.

Brain-behavior correlation analysis

Pearson’s correlation analysis was performed to examine the association between the values of DC or FC and clinical variables such as PSQI, SAS, SDS and insomnia duration. Mean DC or FC values of each spherical ROI with the centroid at its corresponding peak voxel (radius = 5mm) were extracted. The significance level was set at P < 0.05, two-tailed.

**3. Results**

3.1 Clinical Data

Baseline characteristics have previously been described in detail (Yan et al., 2018). Twenty-six PI patients and 28 healthy controls were enrolled. Analysis of demographic variables revealed that no group differences were detected in age, sex, and years of education (all p > 0.05). Significant differences were observed on PSQI, SAS and SDS between two groups (all p < 0.000, two-sample t-tests).

3.2 Degree Centrality Analysis

DC maps of the two groups are shown in Figure 1. The results obtained from the two sample t-test clearly showed highly similar intragroup differences of binary DC between the two groups in several thresholds at r_0_ = 0.10, 0.15, 0.20, 0.25, 0.30, and 0.35. The functional hubs mainly localized in the middle temporal gyrus (MTG), inferior frontal gyrus (IFG), calcarine, and precuneus (S1 Table). For this reason, the study mainly reported the results for DC at r_0_ = 0.25.

The PI patients exhibited a significantly decreased DC in the left IFG and MTG, when compared with the healthy controls (Table 1, Figure 2). The PI patients also demonstrated a significantly increased DC in the right precuneus region.

3.3 Functional Connectivity Analysis

The centered point of the peak t value in brain regions (left IFG, left MTG and right precuneus) that showed significant differences in DC between the PI patients and healthy controls were defined as spherical ROIs (r = 5 mm). We further examined seed-based FC between 3 ROIs and whole brain regions. As compared with healthy controls, decreased FC of the left MTG was found in PI patients mainly located in the left posterior cingulate cortex (PCC) areas (Table 2, Figure 3). When compared with healthy controls, the PI patients exhibited a lateralized increase in FC between the right precuneus and the right lateral occipital cortex (LOC). However, the PI patients did not reveal any suprathreshold clusters between the ROI of left IFG and whole brain regions.

3.4 Correlation Results

The mean DC or FC values were extracted in the five regions (left IFG, left MTG, and right precuneus, left PCC, and LOC) with significant group differences. As shown in Figure 4, the Pearson’s correlation analyses, demonstrated that the reduced DC value of the left IFG was positively correlated with PSQI (r = 0.527, p = 0.006) in PI patients, and was also positively correlated with SAS (r = 0.393, p = 0.038) in healthy controls. Besides, the decreased DC value in the left MTG was positively correlated with SAS (r = 0.400, p = 0.035) and SDS (r = 0.467, p = 0.012) in healthy controls. Finally, the reduced FC between the left MTG and PCC was positively correlated to ISI (r = 0.426, p = 0.003). The decreased DC in the precuneus and its abnormal FC with right LOC had no relationship to any clinical variables.

**Discussion**

In the current study, we investigated the abnormal intrinsic functional hubs and functional whole-brain network in PI patients by a combination of voxel-wise DC and seed-based FC analyses. Using a data-driven approach to investigate the degree of centrality, the results revealed that a set of cortical hubs persisted, including significantly lower DC values in the left inferior frontal gyrus and middle temporal gyrus, and higher DC value in the right precuneus. The seed-based FC analyses described more details about the altered functional networks anchored in these regions. Of note, these intrinsic functional hubs and altered connectivity strength revealed linear correlation with clinical features.

Our results revealed that the IFG is one of the main cortical hubs in the brain network affected by PI. This finding is in line with the previous studies showing the involvement of the prefrontal cortex in insomnia (Nofzinger et al., 2004;Altena et al., 2008;Joo et al., 2013;Gao et al., 2015;Li et al., 2016a). For example, the IFG demonstrated verbal ﬂuency-related brain hypoactivation in chronic insomnia patients that recovered after sleep therapy (Altena et al., 2008). In addition, with the use of low frequency ﬂuctuations (ALFF), Li et al found PI patients showed lower ALFF value in the left IFG, and a negative correlation between the duration of PI and the ALFF value in the left IFG was observed (Li et al., 2016a). Specifically, using graph theoretical analysis, Lu et al observed that healthy participants with insomnia symptom presented reduced regional degree and efficiency in the left IFG compared with subjects without insomnia symptom (Lu et al., 2017). In the present study, we observed diminished DC value in the left IFG, and the DC value was positively correlated with PSQI in PI patients. Hence, our findings might speculate that IFG is a vulnerable region in the pathological process of PI. Interestingly, a positive correlation between the SAS and the DC value in the left IFG was also found in healthy controls. The prefrontal cortex has long been considered to play a vital role in emotional processing (Etkin et al., 2011). Our current study provides evidence that impaired connectivity in the prefrontal cortex, specifically the IFG, might be associated with poor sleep quality under the state of insomnia, while the connectivity in the IFG might be associated with anxiety in healthy subjects without insomnia symptom.

The left MTG demonstrated significantly reduced DC value in PI patients in this study. The MTG is known as a key region during both encoding and retrieval of emotional episodic memories (Fell et al., 2006;Dolcos et al., 2017), and is thought to be involved in dream encoding and recall in both rapid eye movement (REM) and non-REM (NREM) sleep (Cipolli et al., 2017), which is also thought to be involved in the pathology of insomnia (Joo et al., 2013;Li et al., 2016a). Using ALFF algorithm, Li et al observed PI patients showed higher spontaneous regional brain activity in the MTG (Li et al., 2016a). These findings suggested that insomnia is associated with altered MTG function. Structurally, PI patients had smaller volumes of gray matter in the area of MTG (Joo et al., 2013). The brain morphological results further confirmed that functional abnormalities of MTG in insomnia have an anatomical basis. In the current study, the left MTG exhibited decreased FC with the left PCC in PI patients. Previous neuroimaging studies have identified that abnormal FC in the PCC could account for insomnia disorders (Li et al., 2014b). By using positron emission tomography scans, Kay et al examined the relative regional cerebral metabolic (rCMRglc) rate for glucose in PI and controls with a normal sleep pattern during both morning wakefulness and NREM sleep at night, and significant group-by-state interactions in relative rCMRglc were found in the PCC during non-REM sleep (Kay et al., 2016). This finding suggested that insomnia is associated with impaired disengagement of brain regions in the PCC. To supplement this, we found reduced FC between the left MTG and the left PCC may reflect a relationship with insomnia.

The affected brain regions, the MTG and PCC, are considerably overlapped with the default-mode network (DMN), which plays an important role on consciousness modulation (Raichle et al., 2001). Previous neuroimaging studies have pointed out that structural and functional abnormalities in insomnia are related to DMN alterations (Nie et al., 2015;Zhao et al., 2015;Regen et al., 2016;Suh et al., 2016). For instance, decreased structural connectivity between anterior and posterior regions of the DMN in the PI group has been found by using structural MRI. Moreover, decreased structural covariance within the DMN has correlation with higher PSQI scores (Nie et al., 2015). These results indicate that the disrupted DMN may implicate commonly observed sustained sleep difficulties in insomnia. Similarly, a correlation between FC values in left PCC and ISI scores was found in the current study. Our results support the functional disruption of DMN can probably be used to assess the severity of insomnia. Besides, the DC value in the left MTG was positively correlated with SAS and SDS in healthy controls. The DMN is believed to underlie self-reflective processes (Andrews-Hanna et al., 2014), while it is directly proportional to the subject’s anxiety level when performing a task (Raichle, 2015), and resting-state functional connectivity variations has been repeatedly verified in relation to depression (Hamilton et al., 2011). Our findings reveal that, in healthy subjects, higher connectivity density in the left MTG may reflect lower levels of anxiety and depression, although more studies are needed to confirm it.

The precuneus, a part of parietal lobe, is also one of the key regions of DMN, ascending projections to somatosensory, cognitive and visual cortex, and has been proven to be involved in the interwoven network of the self-conscious neural correlates during rest (Cavanna and Trimble, 2006;Utevsky et al., 2014). This result is consistent with selective hypometabolism in the precuneus which has been observed in mental states of decreased or abolished consciousness, such as sleep, drug-induced anesthesia and vegetative states (Cavanna and Trimble, 2006). During wakefulness, cerebral glucose metabolism in the precuneus is at the highest level. While, during slow-wave sleep and REM sleep, the precuneus is one of most deactivated brain areas (Li et al., 2018a). Furthermore, PI patients exhibited decreased spontaneous regional brain activity values in the precuneus (Zhou et al., 2017). These results indicate that the abnormal precuneus functions might influence sleep quality, and our study provides the evidence of disrupted global function in the precuneus.

Moreover, an increase FC between the right precuneus and the right LOC was observed in PI patients. Altered metabolism and spontaneous activity in the occipital cortex had been found in PI patients. The mean occipital g-aminobutyric acid (GABA) level was 33% lower in PI patients than in normal subjects by using single-voxel proton magnetic spectroscopy (Plante et al., 2012). GABA, an inhibitory neurotransmitter, has a role in the etiology and/or maintenance of insomnia (Lu et al., 2006). Decreased GABA in the occipital cortex suggests that increased activity in some neurons in the occipital cortex may result in a hyperarousal state in insomnia. Besides, increased parietal-occipital electroencephalographic (EEG) gamma activity was be found in persons after meditative training during NREM sleep (Ferrarelli et al., 2013). This finding might suppose that parietal-occipital EEG gamma power was a sensitive measure on brain function in sleep. We observed an increase FC between the right precuneus and the right LOC in insomnia. The result shed new light on the pathological mechanism in insomnia.

The limitations of our study are noted below. First, as our study sample is relatively small, the results in this study need further verification in a large sample. Second, this was a cross-sectional study, and it is inadequate to identify the pattern of changes in brain activation. Thus, further longitudinal imaging studies with treatments are needed. Third, the significant association of clinical features and the pattern of changes in brain reported in the present study should be regarded as exploratory in nature due to the fact that no correlation persisted (P < 0.05) after FDR correction for multiple comparisons. Future studies with rigorous multiple testing correction need to be performed. Fourth, fMRI data from wakefulness and a deep sleep state are essential for us to understand how brain function inﬂuences by insomnia. A major methodological constraint is that sleep in a scanner will be difficult to achieve, therefore only waking state fMRI data were obtained in this study. Besides, the affected areas in PI patients in our study are diffuse, and it is difficult to establish their participation in the insomnia disorder. However, a meta-analysis clearly indicated a wide range of brain alterations were presented in insomnia disorder (Tahmasian et al., 2018). Further studies on pathogenic mechanism of insomnia are needed to reveal the specific role of these affected brain regions in insomnia disorder.

In summary, this research used voxel-wise DC and seed-based FC to investigate the intrinsic functional hubs or whole brain functional connection changes in PI patients. The results revealed that PI patients exhibited lower DC values in the left LFG and MTG, and higher DC values in the right precuneus. Decreased FC strength was showed between the left MTG and the left PCC, and increased FC be also found between the right precuneus and the right LOC. Furthermore, reduced DC in left LFG and decreased FC in the left PCC were positively correlated with sleep quality. The findings of this study provide a better understanding of the nature of disconnection in PI patients, which might be helpful to figure out the neurobiological mechanism of insomnia.

**Legends**

Figure 1. Compared to healthy controls, the PI patients exhibited remarkably similar altered DC brain areas in different correlation thresholds (r_0_ = 0.15, 0.2, 0.25, 0.3 and 0.35). The effects are significant at a single voxel p < 0.05, GRF corrected cluster level p < 0.005. The hot (cool) color indicates significantly increased (decreased) DC brain area.

Figure 2. Scatter plot of DC for the significant decreased (increased) clusters between the PI patients and Healthy controls (r0=0.25). The difference between the PI patients and healthy controls based on the DC in the left inferior frontal gyrus (a), left middle temporal gyrus (b), right precuneus (c); Red dots: PI patients; black dots: healthy controls. Error bars represent standard deviation of the mean (** P < 0 .001).

Figure 3. Group differences in seed-based functional connectivity. The seeds were defined as left middle temporal gyrus and right precuneus. Scatter plot of FC for the significant increased or decreased clusters between the PI patients and healthy controls. Red dots: PI patients; black dots: healthy controls. Error bars represent standard deviation of the mean (* P < 0 .05, ** P < 0 .001).

Figure 4. (a) The correlation between the PSQI scores and DC values in the left inferior frontal gyrus; (b) The correlation between SAS scores and DC values in the left inferior frontal gyrus; (c) The correlation between SAS scores and DC values in left middle temporal gyrus; (d) The correlation between SDS scores and DC values in left middle temporal gyrus. (e) The correlation between ISI scores and FC values in left posterior cingulate cortex. Red dots: PI patients; black dots: healthy controls.

Table 1 Significant differences in degree centrality (r_0_ = 0.25) and functional connectivity between two groups.

|  | **Brain regions** | **Side** | **Condition** | **MNI coordinates** | | | **Cluster size** | **Peak t value** |
| --- | --- | --- | --- | --- | --- | --- | --- | --- |
| **Type** |  |  |  | **x** | **y** | **z** |  |  |
| **Degree Centrality** |  |  |  |  |  |  |  |  |
|  | Inferior Frontal Gyrus | Left | PI < HC | -54 | 24 | 15 | 540 | -4.62 |
|  | Middle Temporal Gyrus | Left | PI < HC | -66 | -27 | -9 | 1054 | -4.65 |
|  | Precuneus | Right | PI > HC | 36 | -63 | 27 | 763 | 4.08 |
| **IFG_L-seeded connectivity** | No Suprathreshold clusters | - | - | - | - | - | - | - |
| **MTG_L-seeded connectivity** | Posterior Cingulate Cortex | Left | PI < HC | -3 | -48 | 18 | 944 | -4.35 |
| **Precuneus_R-seeded connectivity** | Lateral Occipital Cortex | Right | PI > HC | 42 | -78 | -6 | 633 | 3.24 |

Abbreviations: IGG, Inferior Frontal Gyrus; MTG, Middle Temporal Gyrus; L(R), left (right) hemisphere.

**Footnotes**

**Contributors** Conceived and designed the experiments: Qing-Quan Liu. Performed the experiments: Jian-Wei Hou, Ping Zhou, Qing-Nan Fu, Jie Zhang, Zhong-Yan Wang. Analyzed the data: Xu Wang, Xue-Rui Wang, Jin-ling Li. Wrote the paper: Chao-Qun Yan, Xu Wang, and Cun-Zhi Liu. All authors approved the final manuscript.

**Funding** This work was supported by Beijing Municipal Science & Technology Commission (China) (ref: Z131100006813002), Beijing Municipal Administration of Hospitals Clinical Medicine Development of Special Funding Support, code: ZYLX201611, and the Fundamental Research Funds for the Central Universities (2017-JYB-JS-018).

**Competing interests** We declare that we have no competing interests.

**Ethics approval** The Research Ethical Committee of Beijing Hospital of Traditional Chinese Medicine Affiliated to Capital Medical University approved the trial.

**Patient consent** Obtained.

**Provenance and peer review** Not commissioned; externally peer reviewed.

**Data Sharing Statement** No additional data are available.
